# Clinical and Radiographic Evaluation of Molar Root–Incisor Malformation (MRIM): A Case Series

**DOI:** 10.1155/crid/9917577

**Published:** 2025-11-28

**Authors:** Ayşegül Karahan, Merve Mısır, Mine Koruyucu

**Affiliations:** Department of Pediatric Dentistry, Faculty of Dentistry, Istanbul University, Istanbul, Turkey

**Keywords:** cervical enamel defects, dental radiography, molar root–incisor malformation, pediatric dentistry, root malformations, tooth abnormalities

## Abstract

**Background:**

Molar root–incisor malformation (MRIM) is a rare dental anomaly characterized by root malformations, abnormal pulp chamber development, and dental developmental defects. Primarily affecting the permanent first molars, MRIM can also involve the primary second molars and permanent maxillary central incisors. The etiology of MRIM remains unclear, though it has been associated with environmental and systemic factors. Early diagnosis and intervention are essential for preventing further complications.

**Cases:**

This report presents five pediatric cases diagnosed with MRIM at our pediatric dentistry clinic between 2016 and 2025. Case 1: A 10-year-old male patient presented for a routine examination. MRIM was detected in Teeth 16, 26, and 36, with enamel depressions on Teeth 11 and 21. No systemic disease or family history of dental anomalies was noted. Case 2: An 8-year-old female patient presented with esthetic concerns due to dental crowding. MRIM affected multiple teeth (16, 26, 36, 46, 13, 23, 11, 21, 31, and 41). The patient had a history of pelvicaliectasis and nephrological follow-up. Case 3: A 12-year-old female patient was referred due to suspected root anomalies. MRIM was observed in all permanent teeth except anterior incisors and third molars. A history of hemolytic uremic syndrome was noted. Case 4: An 11-year-old male patient presented with MRIM involving molars and canines. A neonatal history of severe hypertension and NICU admission was reported. No familial dental anomalies were found. Case 5: A 9-year-old female patient diagnosed with Williams syndrome and other systemic conditions exhibited MRIM in multiple teeth. Structural anomalies such as high-arched palate, ankyloglossia, and deep bite were also observed.

**Conclusion:**

The cases demonstrate the variability of MRIM in pediatric patients, with distinct radiographic and clinical features observed across the cohort. The absence of similar findings in family members suggests that a direct genetic basis is unlikely. Instead, the findings support a possible association between MRIM and systemic conditions, particularly nephrological diseases and complex medical histories. Early and accurate diagnosis, along with close follow-up, is vital in managing MRIM and preventing future complications. A comprehensive medical history and thorough radiographic evaluation of all tooth groups remain essential for accurate diagnosis and treatment planning.

## 1. Introduction

Tooth development is a complex and prolonged biological process shaped by the interaction between the oral ectoderm and mesenchymal cells derived from the cranial neural crest [1]. This process is tightly regulated by cellular and molecular signaling pathways, and any disruption in these mechanisms may result in developmental disturbances, leading to anomalies in the number, shape, or structure of teeth [2, 3].

Molar–incisor malformation (MIM) [4], also referred to as molar root–incisor malformation (MRIM) [5], was first defined in 2014 as a rare dental anomaly primarily affecting the permanent first molars. In some cases, the second primary molars and permanent maxillary central incisors may also be involved. Radiographically, the permanent first molars affected by MRIM exhibit normal crown morphology but are characterized by root malformations such as a narrowed pulp chamber, displaced furcation, and root perforations [6]. In addition, an abnormal mineralized structure, termed the cervical mineralized diaphragm (CMD), has been identified at the level of the cementoenamel junction (CEJ), which has been associated with impaired root development [4–6]. Furthermore, cervical hypoplastic enamel defects may also be observed in the maxillary central incisors affected by MRIM [7].

The clinical and radiographic features of MRIM share morphological similarities with other well-known dentin anomalies; therefore, differential diagnoses should include idiopathic root resorption, regional odontodysplasia, dentin dysplasia Type I (DD-I), and molar–incisor hypomineralization (MIH) [4, 8, 9]. Among these, DD-I presents the most comparable clinical and radiographic features. However, several distinctions are critical for accurate diagnosis. DD-I is a hereditary disorder, typically showing autosomal dominant inheritance and involving the entire dentition [7, 10]. In contrast, MRIM is considered a sporadic anomaly confined to specific teeth, most often the permanent first molars and maxillary central incisors [5]. Radiographically, DD-I is characterized by generalized pulp obliteration and extremely short, blunt roots throughout the dentition, frequently leading to early tooth loss [7]. Conversely, MRIM-affected teeth exhibit normal crown morphology with localized root malformations, such as tapered roots and CMD at the CEJ, without the generalized involvement typical of DD-I [4–6].

Although the exact etiology of MRIM remains unclear, multiple cases have been associated with systemic diseases or the use of prescribed medications during early childhood [4, 7, 9]. The selective involvement of certain teeth and the chronological developmental pattern of MRIM suggest that systemic or environmental factors during early childhood may play a pivotal role in its etiopathogenesis [11].

This study was designed to provide a detailed clinical and radiographic evaluation of patients diagnosed with MRIM in our clinic. In accordance with the existing literature, the patients' medical histories, early-life environmental exposures, and the observed clinical and radiographic findings will be thoroughly analyzed. By investigating potential factors associated with MRIM etiology, this study is aimed at contributing to the existing body of knowledge and offer new insights into the clinical and radiographic characteristics relevant to the diagnostic process.

## 2. Case Presentations

### 2.1. Case 1

**Figure 1:**
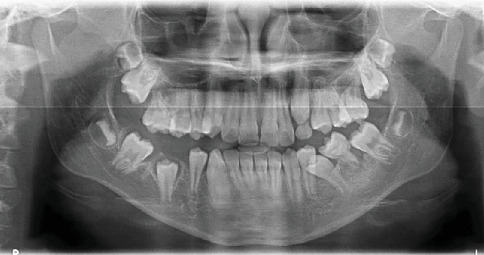
Panoramic radiograph of a male patient showing abnormal root development of Teeth 16, 26, and 36 consistent with molar root–incisor malformation (MRIM). The crown morphology appears normal.

**Figure 2:**
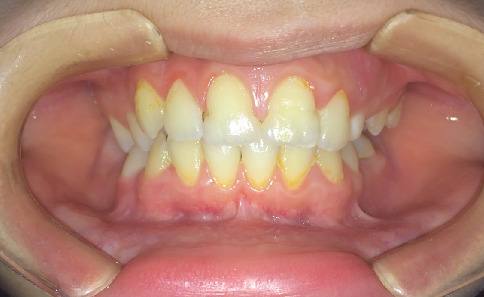
Clinical photograph of Case 1 demonstrating groove-like enamel depressions on the labial surfaces of the maxillary central incisors (Teeth 11 and 21). No crown anomalies are observed in the permanent molars.

In 2016, a 10-year-old male patient presented to the Department of Pediatric Dentistry at Istanbul University Faculty of Dentistry for a routine dental examination. Medical history obtained during the anamnesis revealed no presence of systemic disease or history of preterm birth. A routine panoramic radiograph was taken, which revealed MRIM in the roots of Teeth 16, 26, and 36. It was also noted that Tooth 46 was absent intraorally, and based on the family's report, it had previously been extracted at another clinic due to caries. Intraoral examination showed no crown anomalies in the permanent molars. However, groove-like enamel depressions were observed on the crown surfaces of Teeth 11 and 21, which were also visible in the radiographic images. Radiographic and clinical examinations were subsequently performed on the patient's parents and sibling. All were found to be systemically healthy, and no dental anomalies or MRIM findings were detected. Furthermore, an extended family history was reviewed, and no similar anomalies were reported among relatives on either the maternal or paternal side. The patient had no active dental caries upon clinical examination. Topical fluoride and varnish applications were performed as part of preventive care, and oral hygiene instructions were provided. Radiographic anomalies showing MRIM-affected Teeth 16, 26, and 36 are presented in Figure 1, and intraoral enamel depressions on the maxillary central incisors are illustrated in Figure 2.

### 2.2. Case 2

**Figure 3:**
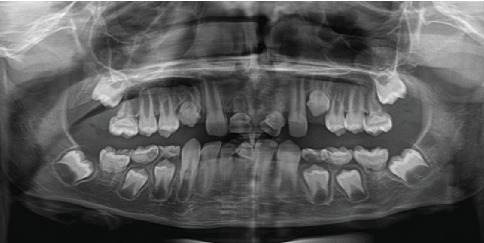
Panoramic radiograph of a female patient (Case 2) demonstrating abnormal root development in multiple teeth (16, 26, 36, 46, 13, 23, 11, 21, 31, and 41) consistent with molar root–incisor malformation (MRIM).

**Figure 4:**
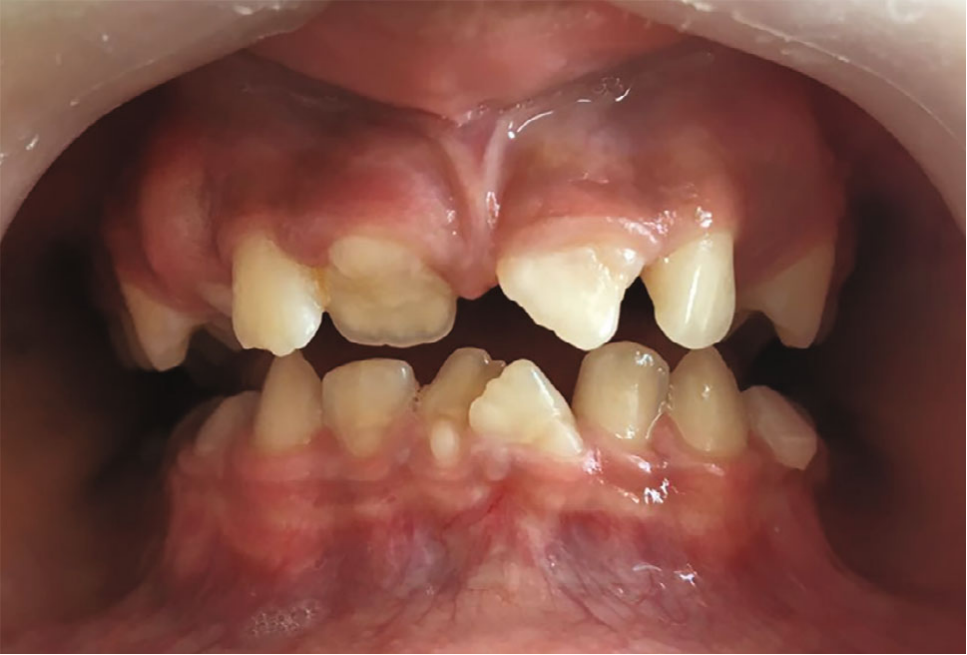
Intraoral photograph of Case 2 illustrating morphological crown anomalies, crowding, and malalignment, particularly affecting the maxillary and mandibular anterior teeth.

In 2016, an 8-year-old female patient presented to our clinic accompanied by her family, expressing esthetic concerns due to dental crowding. Clinical and radiographic examinations revealed the presence of MRIM in Teeth 16, 26, 36, 46, 13, 23, 11, 21, 31, and 41. Intraoral examination showed no morphological anomalies in the crowns of the molars. However, morphological crown anomalies were observed in Teeth 11, 21, 31, and 42. Upon taking the family history, it was determined that none of the patient's three siblings, parents, or extended family members (aunts, uncles, and grandparents from both maternal and paternal sides) had similar dental anomalies. Medical anamnesis revealed that the patient was under nephrological follow-up. According to the patient's medical records, she had been diagnosed with pelvicaliectasis in 2010. Ultrasound findings consistent with this condition showed Grade 1 hydronephrosis localized in the left kidney, along with ureteral dilatation and urolithiasis. In a 2012 ultrasound report, the right kidney appeared normal in size and position, while the left kidney dilation persisted. Follow-up ultrasonography conducted in 2016, when the patient first visited our clinic, demonstrated that the same renal findings continued and additionally revealed the presence of an ovarian cyst in the left ovary. During a follow-up visit in 2018, it was observed that the crown of Tooth #11 had been lost due to structural anomaly, whereas all other teeth were still present. No significant changes were observed in the roots previously diagnosed with MRIM. Panoramic and intraoral views showing MRIM findings in molars, canines, and incisors are presented in Figures 3 and 4.

### 2.3. Case 3

In 2019, a 12-year-old female patient presented to our clinic upon referral due to suspected root anomalies in permanent teeth. Medical history revealed that the patient had been diagnosed with hemolytic uremic syndrome (HUS), a nephrological condition, at the age of 4 and had since been followed by a nephrology department.

Clinical and radiographic examinations revealed MRIM in all permanent teeth except the anterior incisors and third molars. No crown anomalies were noted upon intraoral examination.

The patient's parents and younger sister were also examined both clinically and radiographically, and no similar dental anomalies were observed. Additionally, no history of similar conditions was reported among extended family members.

Since there were no carious lesions or caries-like defects detected in the oral cavity, oral hygiene education and routine follow-up were recommended.

At the follow-up examination conducted in 2025, it was noted that Tooth 13 had failed to erupt due to poor angulation. The roots of the teeth affected by MRIM had not shown any further pathological changes, and the third molar roots were developing normally.

**Figure 5:**
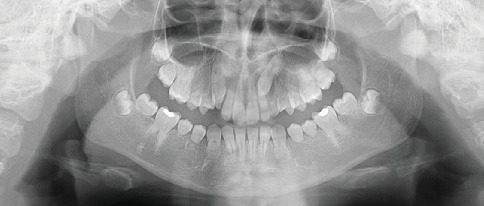
Panoramic radiograph of a female patient (Case 3) revealing abnormal root development consistent with molar root–incisor malformation (MRIM) affecting all permanent teeth except the anterior incisors and third molars. No crown anomalies are visible.

Throughout this 6-year observation period, the patient did not lose any teeth due to MRIM, and no caries formation was observed. Oral hygiene instructions were reinforced at each visit. The panoramic radiograph displaying widespread MRIM involvement across the permanent dentition, excluding the anterior incisors and third molars, is shown in Figure 5.

### 2.4. Case 4

**Figure 6:**
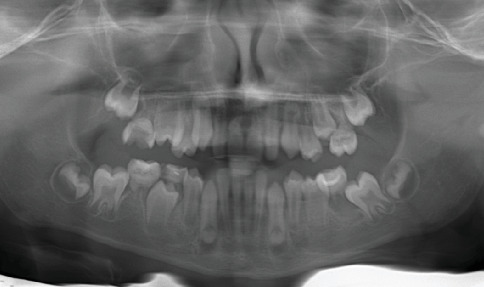
Panoramic radiograph of a male patient (Case 4) showing MRIM-affected teeth, including the permanent first molars (Teeth 16, 26, 36, and 46), canines (Teeth 13, 23, 33, and 43), and maxillary central incisors (Teeth 11 and 21). Abnormal root development and furcation displacement are observed, while crown morphology appears normal.

**Figure 7:**
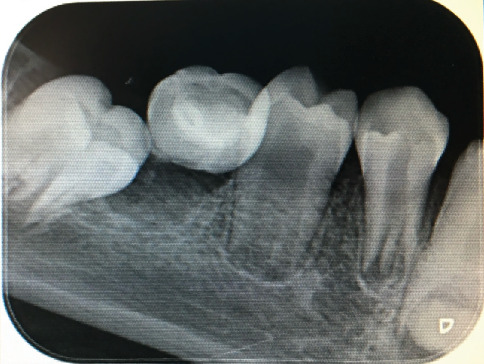
Periapical radiograph of the mandibular right posterior region of Case 4 demonstrating narrowed pulp chamber, atypical furcation position, and short root formation in the first molar (Tooth 46), consistent with MRIM findings.

**Figure 8:**
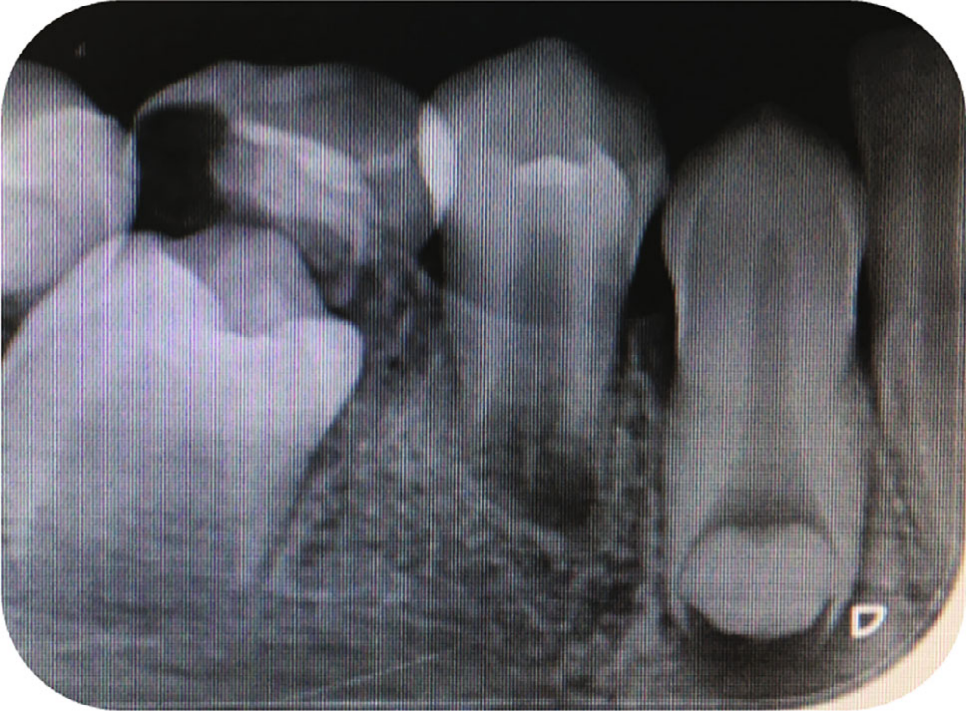
Periapical radiograph of the mandibular canine region of Case 4 demonstrating short, tapered roots with apical constriction and a narrowed pulp chamber, consistent with MRIM findings. The crown morphology appears normal.

In 2019, an 11-year-old male patient presented to our clinic. Clinical and radiographic evaluations revealed the presence of MRIM in Teeth 16, 26, 36, 46, 13, 23, 33, 43, 11, and 21. No crown anomalies were observed during intraoral examination. According to the medical history, the patient had experienced a week-long stay in the neonatal intensive care unit due to severe hypertension during infancy and had not been breastfed. Radiographic and clinical examinations of the patient's parents, twin sibling, and younger sibling revealed no similar MRIM-like or other dental anomalies. Caries detected in several teeth were treated with appropriate restorative procedures. At the follow-up appointment in 2025, all MRIM-affected teeth were still present, and no new carious lesions were found. Oral hygiene instructions were reinforced, and continued preventive care was recommended. Radiographic images depicting MRIM-affected molars and canines are presented in Figures 6, 7, and 8.

### 2.5. Case 5

**Figure 9:**
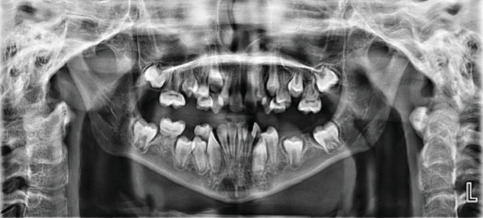
Panoramic radiograph of a 9-year-old female patient (Case 5) diagnosed with Williams syndrome, demonstrating MRIM-affected teeth, including the permanent first molars (Teeth 16, 26, 46) and premolars (Teeth 14 and 24). Abnormal root development with narrowed pulp chambers and irregular furcation areas is evident. The maxillary and mandibular arches show signs of structural anomalies, including tooth crowding and high-arched palate morphology.

In 2025, a 9-year-old female patient presented to our clinic. Clinical and radiographic examinations revealed the presence of MRIM in multiple teeth. According to the medical history provided by the family, the patient was under multidisciplinary medical follow-up due to a complex systemic condition including Williams syndrome, cerebellar tonsillar ectopia (Chiari malformation), single kidney, and associated comorbidities monitored by neurology, cardiology, immunology, endocrinology, and nephrology departments. She had been receiving hormonal injections, held a certificate of severe disability, and was enrolled in special education programs. Family history revealed no known consanguinity and no similar dental or systemic anomalies. The patient was born via uncomplicated vaginal delivery, and previous genetic testing reportedly yielded no definitive findings. Radiographic analysis showed MRIM-affected roots in Teeth 16, 26, 46, 14, and 24, while Tooth 36 had previously been extracted under general anesthesia at another dental facility. Intraoral examination revealed no active carious lesions; however, there were several structural and morphological findings, including tooth crowding, high-arched palate, ankyloglossia, fissured tongue, and deep bite. Extraoral evaluation showed narrow periorbital region, and the pupils were noted to be large and laterally displaced, features compatible with known phenotypes of Williams syndrome. Due to the lack of current restorative treatment needs and significant cooperation difficulties, only oral hygiene instruction was provided to the patient's caregivers, and routine follow-up was advised. Radiographic findings showing MRIM involvement in multiple teeth of the patient diagnosed with Williams syndrome are illustrated in Figure 9.

## 3. Discussion

MRIM was formally recognized as a distinct developmental anomaly in 2014 [4, 5]. Before its definition, similar cases were often misclassified as DD-I or other root malformations due to overlapping features [7]. The key characteristics that distinguish MRIM are the localized involvement of the permanent first molars and occasionally incisors, the presence of a CMD, and the absence of generalized hereditary patterns [4–6, 10]. These features are critical for differentiating MRIM from DD-I, which affects the entire dentition and follows an autosomal dominant inheritance [7, 10]. In our series, the affected teeth and the lack of family history were consistent with MRIM rather than DD-I.

The clinical and radiographic presentation of MRIM shares certain morphological similarities with other well-known dentin anomalies, making differential diagnosis crucial [7]. The condition that most closely resembles MRIM in clinical and radiographic features is DD-I. While DD-I similarly presents with shortened roots and narrowed pulp chambers, it is a hereditary disorder affecting the entire dentition, whereas MRIM is considered a nonhereditary anomaly confined to specific teeth [10]. Notably, some reported cases in the literature exhibit atypical findings inconsistent with classic DD-I and lack a family history of the disorder. These cases may actually represent MRIM that went unrecognized prior to its formal description in the literature [7].

Permanent first molars are the most commonly affected teeth in MRIM, followed by primary second molars and permanent central incisors [11]. Vargo et al. [8] reported that 98.9% of permanent first molars, 39% of primary second molars, and 35.6% of permanent central incisors were affected. Involvement of permanent lateral incisors, canines, first premolars, second molars, and primary first molars has been reported only rarely [7, 8]. Chun et al. [10], in a large case series comprising 64 patients, identified MRIM-associated findings in 15 permanent canines. Similarly, Kim et al. [12] reported MRIM-related features in permanent canines in four out of 38 patients. In our study, MRIM findings were similarly detected in the permanent canines. Specifically, MRIM was diagnosed in the 13 and 23 teeth in Case 2, and in the 13, 23, 33, and 43 teeth in Case 4. These findings are consistent with the reports of Chun et al. and Kim et al., suggesting that MRIM may not be limited to the molar–incisor axis alone. Although there are limited reports in the literature regarding MRIM involvement of canines, both our findings and the large-scale case series of Chun et al. and Kim et al. emphasize the necessity of carefully and comprehensively evaluating all tooth groups, including canines and premolars, during the diagnostic process. The distribution of MRIM-affected teeth according to type and quadrant is summarized in Table 1.

MRIM is a rare dental anomaly characterized by distinctive root malformations [12]. Clinically, the crowns of the affected permanent first molars and primary second molars usually exhibit normal color and morphology, whereas wedge-shaped cervical enamel defects are often observed in incisors and canines, particularly in the cervical third of the crown [13]. Similarly, in our case series, no crown abnormalities were observed in the molars; however, cervical enamel pits and morphological anomalies were detected in the maxillary central incisors of Case 1 and Case 2. Additionally, similar anomalies were identified in the mandibular central and lateral incisors in Case 2.

Radiographically, the most notable feature was cervical constriction at the CEJ, accompanied by thin, tapered, curved, and short roots [14]. In multirooted teeth, one or more roots often demonstrated atypical development, and soft tissue–like nodular formations were occasionally observed between short mesial roots [9]. In some cases, pulp stones were also identified [15]. In our series, distinct morphological changes in root structures were observed in all patients. Narrowed pulp chambers with a slit-like radiographic appearance were noted, and some cases exhibited radiolucent areas resembling CMD.

Although the exact etiology of MRIM remains unclear, various factors have been proposed in the literature. Epigenetic mechanisms [6], systemic conditions associated with the central nervous system such as meningitis and hydrocephalus [14], premature birth [13–16], prenatal abdominal tumors [9], renal diseases [13, 14], urinary tract infections [13], and postnatal staphylococcal infections [17] have been suggested as major medical contributors. These conditions are considered environmental stressors that may disrupt biological processes during the early stages of root development in childhood. Similarly, in our study, most of the cases had a history of significant systemic illnesses during early childhood. Notably, the prominent presence of renal diseases was striking. The occurrence of MRIM alongside nephrological conditions such as pelvic ectasia, HUS, and unilateral renal agenesis suggests a potential relationship between renal pathologies and dental development. It has been reported in the literature that the numerous metabolic and pathophysiological changes associated with chronic kidney disease (CKD) and its treatment can affect the formation, development, and calcification of teeth, with developmental defects of enamel (DDE) observed more frequently in individuals with CKD compared to healthy peers [18–21]. These findings indicate that similar systemic influences may also play a role in the etiology of MRIM. In this context, new hypotheses regarding MRIM pathogenesis may be proposed, involving mechanisms such as developmental stress, inflammation, medication use, or disturbances in mineral metabolism.

Another notable finding in our case series is the co-occurrence of Williams syndrome and MRIM. Williams syndrome is a complex genetic disorder affecting multiple systems, characterized by supravalvular aortic stenosis, developmental delay, neurological abnormalities, endocrine dysfunctions, and craniofacial anomalies [22]. The systemic problems caused by Williams syndrome, especially during the early years of life, could act as environmental stress factors impacting dental development. Considering the critical role of early childhood systemic health history in the etiology of MRIM, systemic effects associated with Williams syndrome may contribute indirectly to the development of MRIM. Although a direct association between Williams syndrome and MRIM has not yet been reported in the literature, the presence of this association in our case highlights the need for further comprehensive clinical studies to explore this relationship.

Importantly, MRIM cannot be explained solely by systemic diseases. The restriction of root malformations to isolated teeth suggests that nongenetic environmental factors and time-sensitive effects may be involved. To date, no specific genetic mutation has been identified as being associated with MRIM [14].

Lee et al. [6] suggested that deviations in signaling pathways involved in tooth development, early-life systemic conditions, and certain medications may contribute to the development of MRIM. It has also been proposed that external factors during critical periods of root development could influence tooth morphology through mechanisms beyond direct genetic alterations. For example, in a case reported by Choi et al. [15], MRIM was observed in only one of a pair of identical twins, while no dental anomalies were detected in the other twin. Similarly, in our study, no abnormalities were found in the monozygotic twin of the patient diagnosed with MRIM in Case 4. These observations indicate that factors such as environmental influences, systemic illnesses, and individual biological responses may have a role in the pathogenesis of MRIM.

In conclusion, our findings suggest that the etiology of MRIM cannot be attributed to a single cause. Rather, systemic diseases, environmental stressors, and patient-specific susceptibilities appear to interact and collectively contribute to its development. Particularly, systemic illnesses during early childhood seem to play a central role in the manifestation of MRIM. Therefore, careful examination of all tooth groups, along with thorough medical history collection, is essential for improving diagnostic accuracy and understanding the clinical characteristics of MRIM.

## 4. Conclusion

In light of the complex and multifactorial etiology of MRIM, it is crucial for clinicians to obtain comprehensive medical histories and to perform meticulous radiographic evaluations of all tooth groups in order to ensure an accurate diagnosis and to optimize patient management.

## Figures and Tables

**Table 1:** Distribution of MRIM-affected teeth across cases.

**Case**	**Permanent molars**	**Primary molars**	**Central incisors**	**Lateral incisors**	**Canines**	**Premolars**
Case 1	16, 26, 36	—	11, 21	—	—	—
Case 2	16, 26, 36, 46	—	11, 21, 31, 41	42	13, 23	—
Case 3	—	—	—	—	13, 23, 33, 43	—
Case 4	16, 26, 36, 46	—	11, 21	—	13, 23, 33, 43	—
Case 5	16, 26, 46	—	—	—	—	14, 24

## Data Availability

The data supporting the findings of this study are available from the corresponding author upon reasonable request.
